# Empagliflozin rescues pro-arrhythmic and Ca^2+^ homeostatic effects of transverse aortic constriction in intact murine hearts

**DOI:** 10.1038/s41598-024-66098-7

**Published:** 2024-07-08

**Authors:** Qiang Wen, Rui Zhang, Kejun Ye, Jun Yang, Hangchuan Shi, Zhu Liu, Yangpeng Li, Ting Liu, Shiyu Zhang, Wanpei Chen, Jingjing Wu, Weichao Liu, Xiaoqiu Tan, Ming Lei, Christopher L.-H. Huang, Xianhong Ou

**Affiliations:** 1 Department of Cardiology, Union Hospital, Tongji Medical College, Huazhong University of Science and Technology, 1277 Jiefang Rd, Wuhan, 430022 Hubei Province China; 2 Key Laboratory of Medical Electrophysiology of Ministry of Education, Institute of Cardiovascular Research, Department of Cardiology of the Affiliated Hospital, Southwest Medical University, 1 Xianglin Rd, Luzhou, 646000 Sichuan Province China; 3 Department of Clinical & Translational Research, University of Rochester Medical Center, 265 Crittenden Blvd, Rochester, NY 14642 USA; 4 Department of Public Health Sciences, University of Rochester Medical Center, 265 Crittenden Blvd, Rochester, NY 14642 USA; 5 Department of Pharmacology, University of Oxford, Mansfield Road, Oxford, OX1 3QT UK; 6 Physiological Laboratory, University of Cambridge, Downing Street, Cambridge, CB2 3EG UK; 7 Department of Biochemistry, University of Cambridge, Tennis Court Road, Cambridge, CB2 1QW UK; 8 State Key Laboratory for Chemistry and Molecular Engineering of Medicinal Resources, Guangxi Normal University, 15 Yucai Rd, Guilin, 541004 Guangxi Province China

**Keywords:** Empagliflozin, Transverse aortic constriction (TAC), Heart failure, Ca^2+^ homeostasis, Action potential duration, Ventricular arrhythmia, Physiology, Systems biology, Cardiology

## Abstract

We explored physiological effects of the sodium-glucose co-transporter-2 inhibitor empagliflozin on intact experimentally hypertrophic murine hearts following transverse aortic constriction (TAC). Postoperative drug (2–6 weeks) challenge resulted in reduced late Na^+^ currents, and increased phosphorylated (p-)CaMK-II and Nav1.5 but not total (t)-CaMK-II, and Na^+^/Ca^2+^ exchanger expression, confirming previous cardiomyocyte-level reports. It rescued TAC-induced reductions in echocardiographic ejection fraction and fractional shortening, and diastolic anterior and posterior wall thickening. Dual voltage- and Ca^2+^-optical mapping of Langendorff-perfused hearts demonstrated that empagliflozin rescued TAC-induced increases in action potential durations at 80% recovery (APD_80_), Ca^2+^ transient peak signals and durations at 80% recovery (CaTD_80_), times to peak Ca^2+^ (TTP_100_) and Ca^2+^ decay constants (Decay_30–90_) during regular 10-Hz stimulation, and Ca^2+^ transient alternans with shortening cycle length. Isoproterenol shortened APD_80_ in sham-operated and TAC-only hearts, shortening CaTD_80_ and Decay_30–90_ but sparing TTP_100_ and Ca^2+^ transient alternans in all groups. All groups showed similar APD_80_, and TAC-only hearts showed greater CaTD_80_, heterogeneities following isoproterenol challenge. Empagliflozin abolished or reduced ventricular tachycardia and premature ventricular contractions and associated re-entrant conduction patterns, in isoproterenol-challenged TAC-operated hearts following successive burst pacing episodes. Empagliflozin thus rescues TAC-induced ventricular hypertrophy and systolic functional, Ca^2+^ homeostatic, and pro-arrhythmogenic changes in intact hearts.

## Introduction

Sodium-glucose co-transporter 2 inhibitors (SGLT2i) have been reported to reduce mortality and re-hospitalization in heart failure (HF) therapy in the large randomized, double blind DAPA-HF and EMPEROR-Reduced clinical trials, and the CANVAS Program^1–3^. However, their introduction in type 2 diabetic (T2DM) patients did not reduce incidences of ventricular arrhythmias and sudden cardiac death in patients with newly diagnosed^4^, and established T2DM whether in the presence or absence of HF or chronic renal disease^5^. Nevertheless, post hoc analysis of the DAPA-HF study reported that the SGLT2i dapagliflozin reduced risks of serious ventricular arrhythmias, cardiac arrest, or sudden death in HF patients^6^ suggesting particular SGLT2i electrophysiological actions.

Recent experimental studies exploring actions of SGLT2i drugs in metabolic and ischemic experimental cardiac models at the cellular level complement these clinical findings. These reported that, in common with dapagliflozin, empagliflozin shortened cardiomyocyte action potential durations (APD) in experimental rats with diet or streptozotocin induced metabolic syndrome or diabetes^7,8^. In isolated ventricular cardiomyocytes from diabetic rats, empagliflozin reduced the late Nav1.5 currents (*I*_NaL_)^8^ previously associated with pro-arrhythmic tendency in other situations^9,10^. It also attenuated remodeling changes in Ca^2+^ homeostasis in cardiomyocytes from diabetic rats, db/db mice subject to high-fat-diets, and rabbit ischemia models^8,11,12^. Empagliflozin also downregulated Ca^2+^/calmodulin-dependent protein kinase II (CaMK-II) activity in db/db^11^ and TAC-HF mice^13^. This would reduce the actions of CaMK-II in phosphorylating Nav1.5, increasing *I*_NaL_, as well as in phosphorylating Ca^2+^ homeostatic proteins including ryanodine receptor type II (RyR2) and phospholamban (PLN)^8,11,12,14,15^. These cellular findings were in keeping with findings in intact diabetic hearts of shortened electrocardiographic QTc intervals^7,8^, effects on diastolic function^11,16^, and possible ventricular anti-arrhythmic actions associated with ischemic cardiac remodeling^12,17,18^, that could involve hypertrophic changes, at the whole organ level^19^.

There are fewer available studies on SGLT2i actions in hypertensive mice under conditions of HF following transverse aortic constriction (TAC) as opposed to mice under metabolic and ischemic stress. Furthermore these were conducted largely at the cellular rather than the systems level. For example, empagliflozin reduced *I*_NaL_, remodeling of Ca^2+^ homeostasis and CaMK-II activity in ventricular cardiomyocytes from mice following TAC^13,20^. The present complementary experiments test the hypothesis that empagliflozin exerts actions at the *intact* heart level under conditions of TAC-induced HF in accord with predictions from its reported effects at the cellular level. We first replicated the previous reported changes in *I*_NaL_ and phosphorylated (p-)CaMKII activity, under our particular experimental conditions. We then demonstrated positive actions on indicators of cardiac hypertrophy and systolic function within the same hearts. This complemented previous findings in rats with mineralocorticoid-induced hypertension^21^, as well as recent suggestions from cardiomyocyte-level findings implicating electrophysiological effects in SGLT2i cardioprotective actions^22^. Finally we demonstrated actions on hypertrophic and contractile changes and on action potential waveform, intracellular Ca^2+^ homeostasis and ventricular arrhythmogenicity. These findings at the organ level using intact Langendorff-perfused murine hearts were obtained by applying echocardiographic methods and dual wavelength cardiac optical mapping established as applicable to the present studies at the level of intact hearts.

## Results

### Empagliflozin actions on Na^+^ current, and CaMK-II, Nav1.5 and Na^+^/Ca^2+^ exchanger protein expression

The initial controls first confirmed previously reported empagliflozin actions on *I*_NaL_ at their adopted 10 μM concentration. Studies were made in whole cell patch clamped CHO cell lines stably expressing human Nav1.5 channel α-subunits. ATX-II (10 nM) was used to enhance *I*_NaL_. The magnitude of *I*_NaL_ was initially measured as the integral between the baseline and current trace in the 10–40 ms interval following the onset of voltage steps from the − 120 mV resting to a − 15 mV membrane potential. Experiments exploring effects of ATX-II concentration on Na^+^ currents (Supplementary Fig. S1(i)) demonstrated correspondingly increased *I*_NaL_ relative to measurements made in the absence of ATX-II (Supplementary Fig. S1(ii)) giving an EC_50_ of 8.86 ± 0.01 nM (N = 5). These changes took place in an absence of changes in initial, peak Na^+^ current, *I*_NaT_ (Supplementary Fig. S1(iii)).

**Figure 1:**
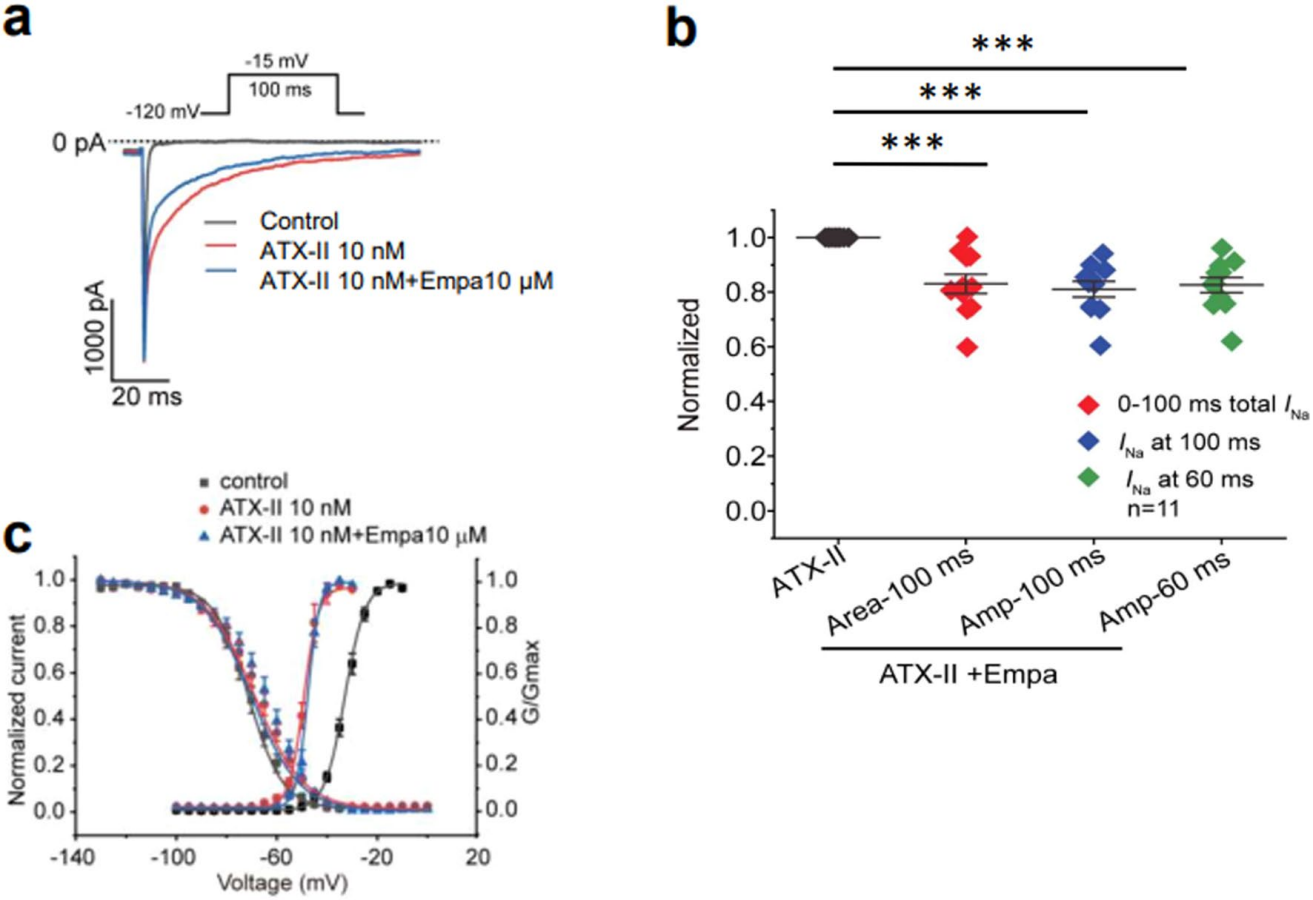
Empagliflozin decreases ATX-II induced *I*_NaL_ while sparing Nav1.5 gating in Nav1.5 expressing CHO cells. (**a**) Na^+^ currents under control conditions and following addition of 10 nM ATX-II and 10 nM ATX-II + 10 μM empagliflozin. (**b**) Normalized inward Na^+^ current measured at 60 ms and 100 ms (designated Amp-60 ms/100 ms) and from the area under the curve between 0 and 100 ms poststimulation (designated Area-100 ms). N = 11 for each group. One way ANOVA with Holm-Sidak’s tests for post hoc multiple comparisons, ***P < 0.001. (**c**) Steady-state Nav1.5 activation data (right curves) obtained using 200 ms duration test steps made to test potentials between − 120 and + 60 mV at 10 mV increments. Steady-state inactivation data (left curves) obtained by a double-pulse protocol imposing a series of 200 ms duration conditioning depolarizing pulses to voltages between − 120 and + 60 mV with 10 mV increments followed by a 300 ms duration test pulse to a fixed voltage of − 30 mV. ATX-II, *anemonia sulcata* toxin II; Empa, empagliflozin; *I*_NaL_, late sodium current.

The effects of empagliflozin on *I*_NaL_ were then examined using the 10 nM ATX-II concentration. Na^+^ currents were compared before (Fig. 1a) and following administration of 10 nM ATX-II and 10 nM ATX-II + 10 μM empagliflozin. Empagliflozin reduced *I*_NaL_, whether quantified by the current at 60 ms and 100 ms, or by the integral of the *I*_NaL_ trace between 0 and 100 ms following stimulation (Fig. 1b). In contrast, empagliflozin did not alter the gating properties of Nav1.5 currents obtained in the presence of ATX-II (Fig. 1c). Thus, addition of ATX-II itself negatively shifted the activation half maximal voltages, *V*_1/2_ by ~ 16 mV (*P* < 0.05; one way ANOVA with Holm-Sidak’s tests for post hoc multiple comparisons). However it spared the steepness factors *k* (*P* > 0.05: before ATX-II: *V*_1/2_ = − 32.57 ± 0.28 mV, *k* = 4.13 ± 0.13 mV (N = 17); in the presence of ATX-II: *V*_1/2_ = − 48.81 ± 0.28 mV, *k* = 3.01 ± 0.16 mV (N = 5); Fig. 1a,c). It also left inactivation parameters unchanged (*P* > 0.05: before ATX-II: *V*_1/2_ = − 71.21 ± 0.59 mV, *k* = 6.81 ± 0.22 mV (N = 17); in the presence of ATX-II: *V*_1/2_ = − 69.04 ± 0.81 mV, *k* = 9.73 ± 0.48 mV (N = 7)). Further addition of empagliflozin produced no further actions on either activation (*V*_1/2_ = − 47.69 ± 0.42 mV, *k* = 2.29 ± 0.22 (N = 5); activation* V*_1/2_ significantly different from control but not in the presence of ATX-II) or inactivation variables (*V*_1/2_ = − 70.08 ± 1.44 mV, *k* = 9.62 ± 0.64 (N = 6); Fig. 1c).

**Figure 2:**
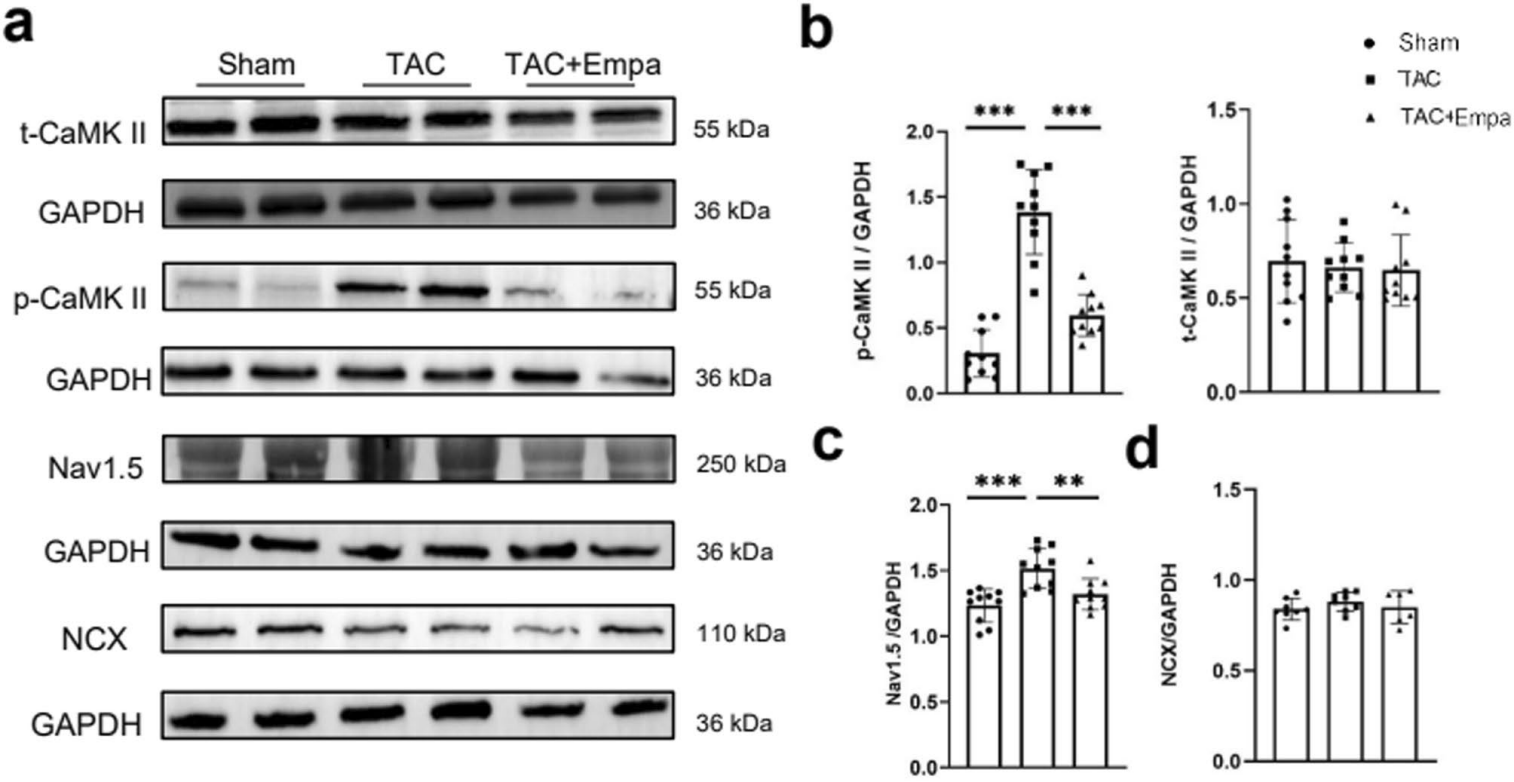
Effect of empagliflozin on CaMK-II phosphorylation and Nav1.5 and NCX expressions. Western blot determinations of protein expression in sham-operated, TAC-only and TAC + empagliflozin (Empa) groups. (**a**) Images of total CaMK-II, phosphorylated CaMK-II, Nav1.5 and NCX. (**b**–**d**) Semi-quantitative analysis of expression results for t/p-CaMK-II (**b**), Nav1.5 (**c**) and NCX (**d**) with one way ANOVA with Holm-Sidak’s tests for post hoc multiple comparisons. **P < 0.01, ***P < 0.001. t/p-CaMK-II, total/phosphorylated calmodulin-dependent protein kinase II; Nav1.5, sodium channel 1.5; NCX, Na^+^/Ca^2+^ exchanger; GAPDH, glyceraldehyde-3-phosphate dehydrogenase. Original Western blot results shown in supplementary file 2.

Secondly, total (t)-CaMK-II, phosphorylated (p)-CaMK-II, Nav1.5 and Na^+^/Ca^2+^ exchanger (NCX) protein expressions were compared between sham-operated, TAC-only and TAC + empagliflozin hearts (Fig. 2a). Higher levels of both p-CaMK-II and Nav1.5 were observed in the TAC-only compared to both sham-operated and TAC + empagliflozin groups (*P* < 0.001 in both cases; *P* < 0.001 and 0.01, respectively; Fig. 2b,c). t-CaMK-II and NCX protein expression contrastingly were indistinguishable between groups (Fig. 2b,d) [See also Supplementary File S2].

### Empagliflozin rescues left ventricular functional and anatomical cardiac remodeling in mice with TAC-induced HF

**Figure 3:**
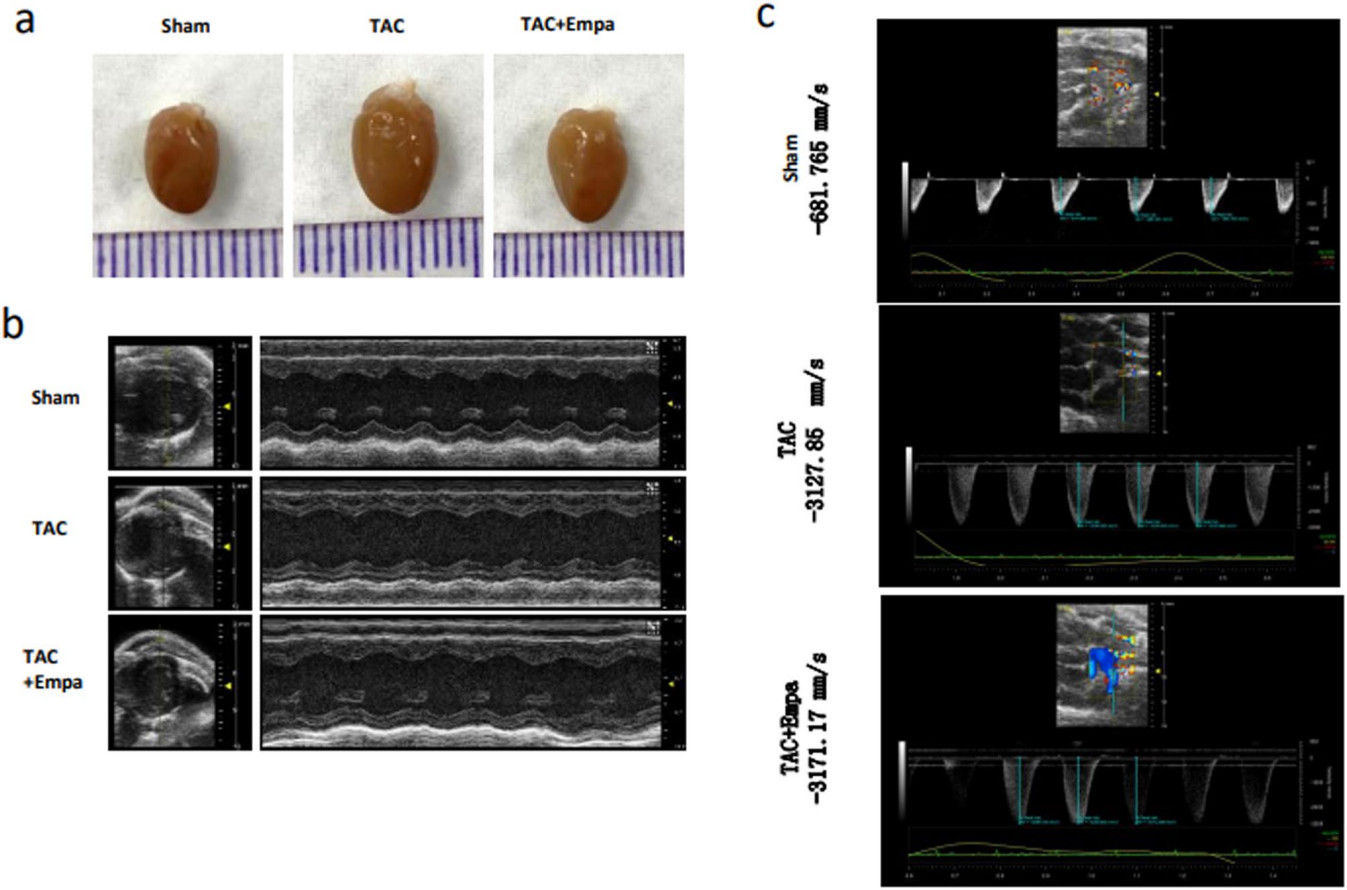
Empagliflozin rescues left ventricular remodeling in TAC-HF mice. (**a**,**b**) Representative heart images (mm scale bar) (**a**) and typical images from M type echocardiography (**b**) from animals in the sham-operated, TAC-only and TAC + Empagliflozin (Empa) groups after 4 weeks of treatment. (**c**) blood flow velocity (mm/sec) determinations across the aortic constriction.

**Figure 4:**
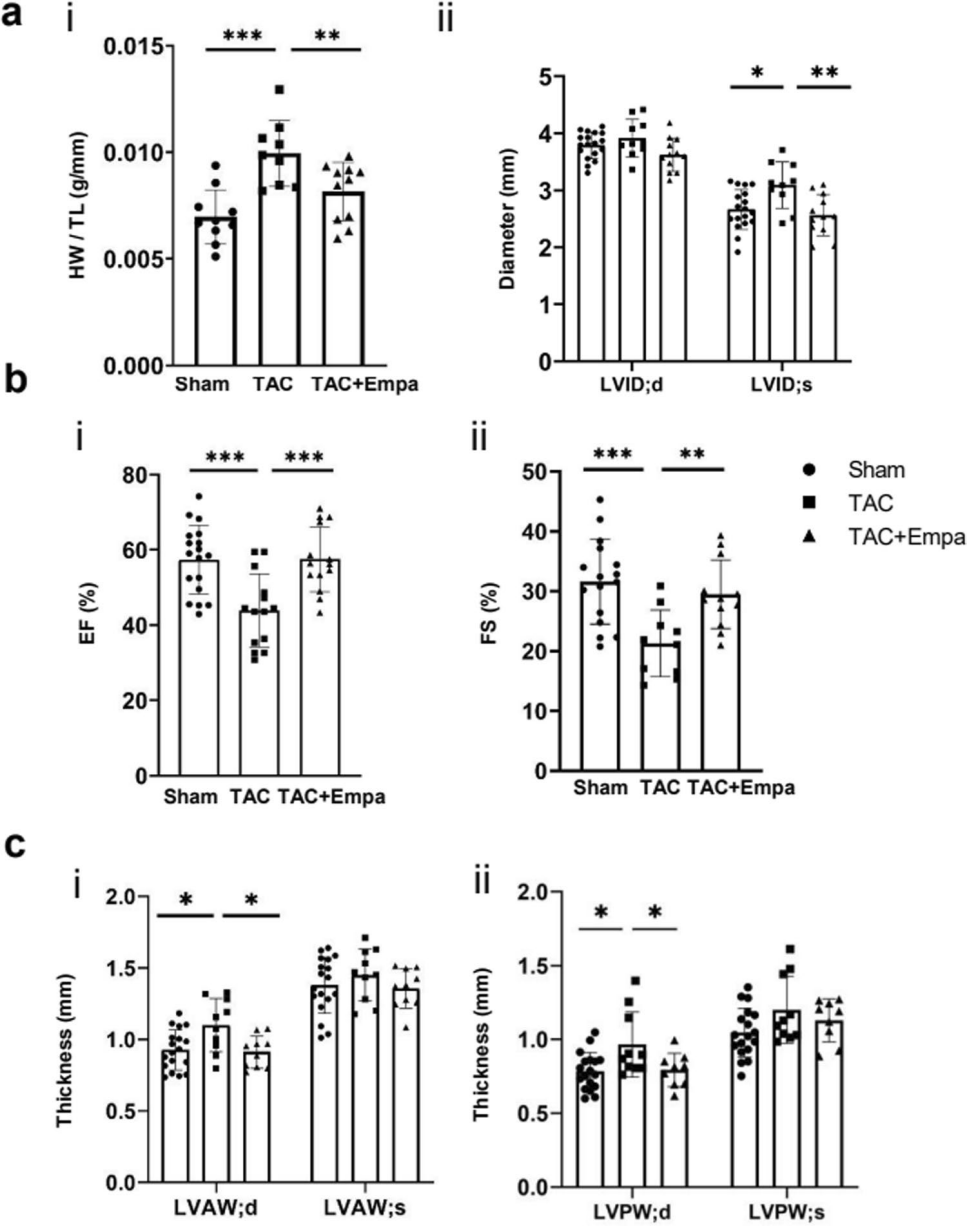
Quantifications of empagliflozin rescue of left ventricular remodeling in TAC-HF mice. (**a**) Heart weight/tibial length (HW/TL) and left ventricular internal diameter ratios. (**b**,**c**) Indices for (**b**) cardiac function: ejection fraction (EF) (i) and fractional shortening (FS) (ii) results; (**c**) structural remodeling: left ventricular diastolic and systolic anterior (i) and posterior (ii) wall thicknesses. *P < 0.05, **P < 0.01, ***P < 0.001, one way ANOVA with Holm-Sidak’s tests for post hoc multiple comparisons. TAC, transverse aortic constriction; Empa, empagliflozin; HW/TL, heart weight/tibia length; LVID, left ventricular internal diameter; EF, ejection fraction; FS, fractional shortening; LVAW, left ventricular anterior wall; LVPW, left ventricular posterior wall; d, diastolic; s, systolic.

Figure 3 exemplifies (a) cardiac appearances, (b) M-type echocardiographic images and (c) trans-aortic flow velocities in hearts subject to sham operations, TAC only and TAC + empagliflozin hearts subject to 4 weeks of empagliflozin therapy begun after 2 weeks following the TAC operation. The TAC-only group showed enlarged hearts compared to the sham-operated group and increased flow velocities. Quantification of these results (Fig. 4) revealed that the TAC-only hearts showed significantly different heart weight/tibial length ratios (HW/TL; *P* < 0.001) and left ventricular internal systolic though not diastolic diameters (*P* < 0.05) from sham-operated hearts. These changes were relieved by empagliflozin (*P* < 0.01 in both cases) (Fig. 4a(i) and (ii)). TAC-only hearts also showed compromised systolic function indicators, quantified as ejection fractions (EF) and fractional shortening (FS), relative to sham-operated hearts (43.88 ± 2.58% vs. 57.36 ± 2.16% for EF, *P* < 0.001; 21.28 ± 1.76% vs. 31.59 ± 1.78% for FS, *P* < 0.001). These were restored by empagliflozin (57.52 ± 2.30% vs. 43.88 ± 2.58% for EF, *P* < 0.001; 29.48 ± 1.66% vs. 21.28 ± 1.76% for FS, *P* < 0.01, Fig. 4b). Finally, TAC-only hearts showed a cardiac remodeling likely resulting from increased intraventricular pressures. They showed greater diastolic anterior and posterior wall thicknesses than sham-operated hearts. This effect was also attenuated by empagliflozin treatment (anterior: 0.93 ± 0.03 mm vs. 1.10 ± 0.06 mm vs. 0.91 ± 0.04 mm for the sham-operated, TAC-only and TAC + empagliflozin groups, *P* < 0.05; posterior: 0.79 ± 0.03 mm vs. 0.97 ± 0.07 mm vs. 0.79 ± 0.04 mm for the sham-operated, TAC-only and TAC + empagliflozin groups, *P* < 0.05, Fig. 4c).

### Empagliflozin rescues electrophysiological remodeling associated with TAC-induced HF

**Figure 5:**
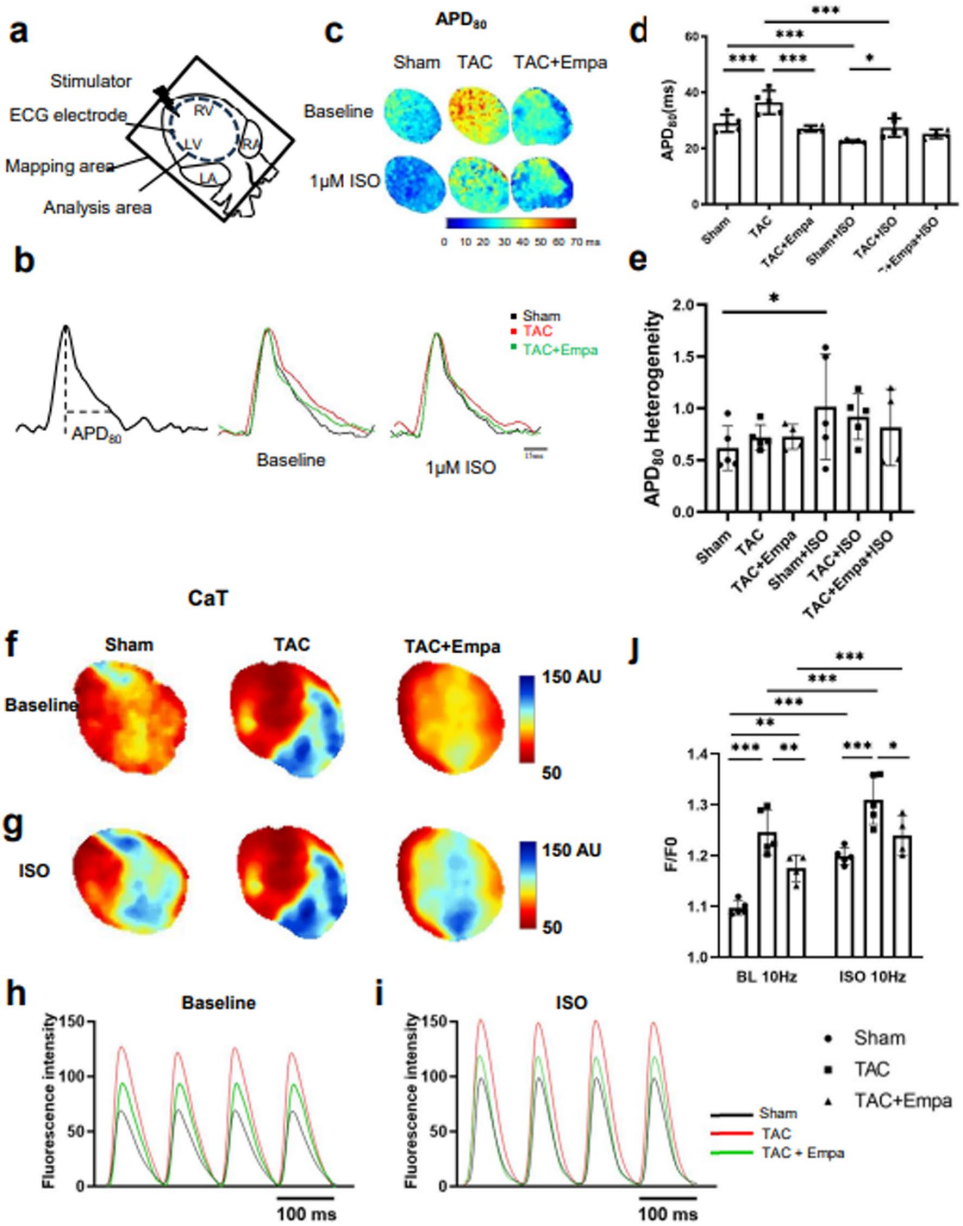
Mapping of electrophysiological and Ca^2+^ homeostatic activity in sham-operated, TAC-only and TAC + empagliflozin hearts. (**a**) Schematic diagram summarizing configuration of dual optical mapping recording system including mapping areas. In these experiments hearts were studied at a regular stimulation frequency of 10 Hz. (**b**–**e**) Action potential (AP) studies: (**b**) Action potential (AP) traces: AP trace illustrating determination of APD_80_ (left) and typical AP traces under baseline conditions (middle) and following 1 μM isoproterenol (ISO) challenge (right) from sham-operated, TAC-only and TAC + empagliflozin groups (colour-coded). (**c**) Typical APD_80_ maps for sham-operated, TAC-only and TAC + empagliflozin groups obtained under baseline conditions and following 1 μM isoproterenol challenge. (**d**,**e**) Summary of APD_80_ (**d**), and APD_80_ heterogeneity results (**e**). (**f**–**j**) Studies of Ca^2+^ signals: (**f**,**g**) typical peak CaT maps from sham-operated, TAC-only and TAC + empagliflozin groups before (**f**) and following isoproterenol challenge (**g**). (**h**,**i**) corresponding Ca^2+^ signal (CaT) traces. (**j**) peak *F*/*F*_0_ values in sham-operated, TAC-only and TAC + empagliflozin groups before (left) and following 1 μM isoproterenol challenge (right). N = 5 for sham-operated and TAC-only groups and N = 4 for TAC + empagliflozin group. *P < 0.05, **P < 0.01, ***P < 0.001, two way ANOVA with Holm-Sidak’s tests.

The effects of empagliflozin on action potential (AP) and Ca^2+^ transient (CaT) changes following TAC were assessed employing dual optical mapping recordings. The Langendorff-perfused hearts were regularly stimulated at 10 Hz before and following isoproterenol (1 μM) challenge. Figure 5a schematizes the optical mapping and ECG monitoring configurations. Mean action potential durations at 80% recovery (APD_80_) were in each case measured from the AP peak. This was determined by averaging component pixel AP waveforms both over the sampled field of view (Fig. 5a,c) and through such values obtained over 30 beats (Fig. 5b). The mean AP heterogeneities were obtained from the difference between APD_80_ values at the 95th and 5th percentile, normalized to the APD_80_ value at the 50th percentile, similarly averaged over 30 beats. Similar averaging procedures were used to derive optical readouts of intracellular Ca^2+^ changes in the subsequent experiments.

Both the AP traces (Fig. 5b) and the maps of action potential duration at 80% recovery (APD_80_) (Fig. 5c) demonstrated APD_80_ prolongation in TAC-only relative to sham-operated groups (See Table 1 for detailed values). Two way ANOVA of APD_80_ results (Fig. 5d) demonstrated independent (*P* < 0.01 and *P* < 0.0001) and interacting effects (*P* < 0.05) of the different interventions involving sham-operated, TAC-only or TAC + empagliflozin groups, and the presence or absence of isoproterenol. Post hoc Holm-Sidak tests revealed: (a) greater APD_80_ in TAC-only than sham-operated hearts whether isoproterenol was absent or present (*P* < 0.001 and 0.05 respectively). (b) Empagliflozin reduced these effects of TAC before (*P* < 0.001) but not following (*P* > 0.05) isoproterenol treatment. (c) Isoproterenol treatment shortened APD_80_ in sham-operated (*P* < 0.001) and TAC-only (*P* < 0.001), but not TAC + empagliflozin groups. Contrastingly, two way ANOVA demonstrated independent effects (*P* < 0.01) of isoproterenol challenge, but not the different interventions, or any interacting effects, on APD_80_ heterogeneity (Fig. 5e). Holm-Sidak tests did not reveal pairwise differences involving sham-operated or TAC-only groups, whether in the presence or absence of empagliflozin challenge, whether isoproterenol was present or absent, apart from isoproterenol increasing APD_80_ heterogeneity in the sham-operated group..

**Table 1:** Physiological parameters (means ± SEM) following TAC before and following empagliflozin in the absence and presence of empagliflozin.

	Isoproterenol absent			Isoproterenol present		
	Sham-operated (N = 5)	TAC-only (N = 5)	TAC + empagliflozin (N = 4)	Sham-operated (N = 5)	TAC-only (N = 5)	TAC + empagliflozin (N = 4)
APD80 (ms)	28.95 ± 1.384	36.42 ± 1.873	27.05 ± 0.561	21.51 ± 0.897	27.42 ± 1.471	25.19 ± 0.811
APD80 heterogeneity	0.616 ± 0.0958	0.720 ± 0.0546	0.725 ± 0.0603	1.016 ± 0.2274	0.920 ± 0.0987	0.817 ± 0.1856
*F*/*F*_0_	1.097 ± 0.00621	1.246 ± 0.0197	1.175 ± 0.0131	1.199 ± 0.0074	1.309 ± 0.0215	1.239 ± 0.0195
CaTD_80_ (ms)	56.83 ± 1.027	60.62 ± 0.614	56.55 ± 1.146	46.07 ± 0.609	51.06 ± 0.810	48.38 ± 0.330
CaTD_80_ heterogeneity	0.179 ± 0.0254	0.306 ± 0.0643	0.148 ± 0.00718	0.187 ± 0.0316	0.364 ± 0.0884	0.139 ± 0.00648
TTP_100_ (ms)	21.87 ± 0.805	25.51 ± 0.573	18.49 ± 0.596	19.54 ± 0.593	23.68 ± 0.692	17.32 ± 0.745
Decay_30–90_ (ms)	51.29 ± 1.666	56.13 ± 1.919	42.69 ± 1.772	33.17 ± 1.186	40.77 ± 2.660	30.98 ± 0.688

### Empagliflozin rescues intracellular Ca^2+^ homeostatic changes associated with TAC-induced HF

The effects of empagliflozin on intracellular Ca^2+^ handling were explored by analysing CaT transients mapped in sham-operated, TAC-only and TAC + empagliflozin ventricles. Traces of the respective mean Ca^2+^ transients (Fig. 5h,i) with peak values *F* normalized to their baseline values *F*_0_, *F*/*F*_0_ were then obtained (Fig. 5j). These peak values showed parallel changes with each intervention before (Fig. 5f) and following (Fig. 5g) isoproterenol challenge. Two way ANOVA demonstrated independent (*P* < 0.001 and *P* < 0.000001) and interacting (*P* < 0.05) effects of the different interventions and the presence or otherwise of isoproterenol challenge. Holm-Sidak tests demonstrated: (a) greater *F*/*F*_0_ in both the absence and presence of isoproterenol in TAC-only than sham-operated groups in the absence of empagliflozin (*P* < 0.00001 and < 0.001); (b) Empagliflozin then reduced the latter effect in the TAC-operated groups (*P* < 0.01 and < 0.05). (c) This left significant *F*/*F*_0_ differences from the sham-operated group in the absence (*P* < 0.01) but not the presence of isoproterenol challenge (*P* > 0.05). Finally, (d) isoproterenol itself increased *F*/*F*_0_ in sham-operated (*P* < 0.000001) and TAC-only animals whether in the absence (*P* < 0.0001) or presence (*P* < 0.0001) of empagliflozin.

**Figure 6:**
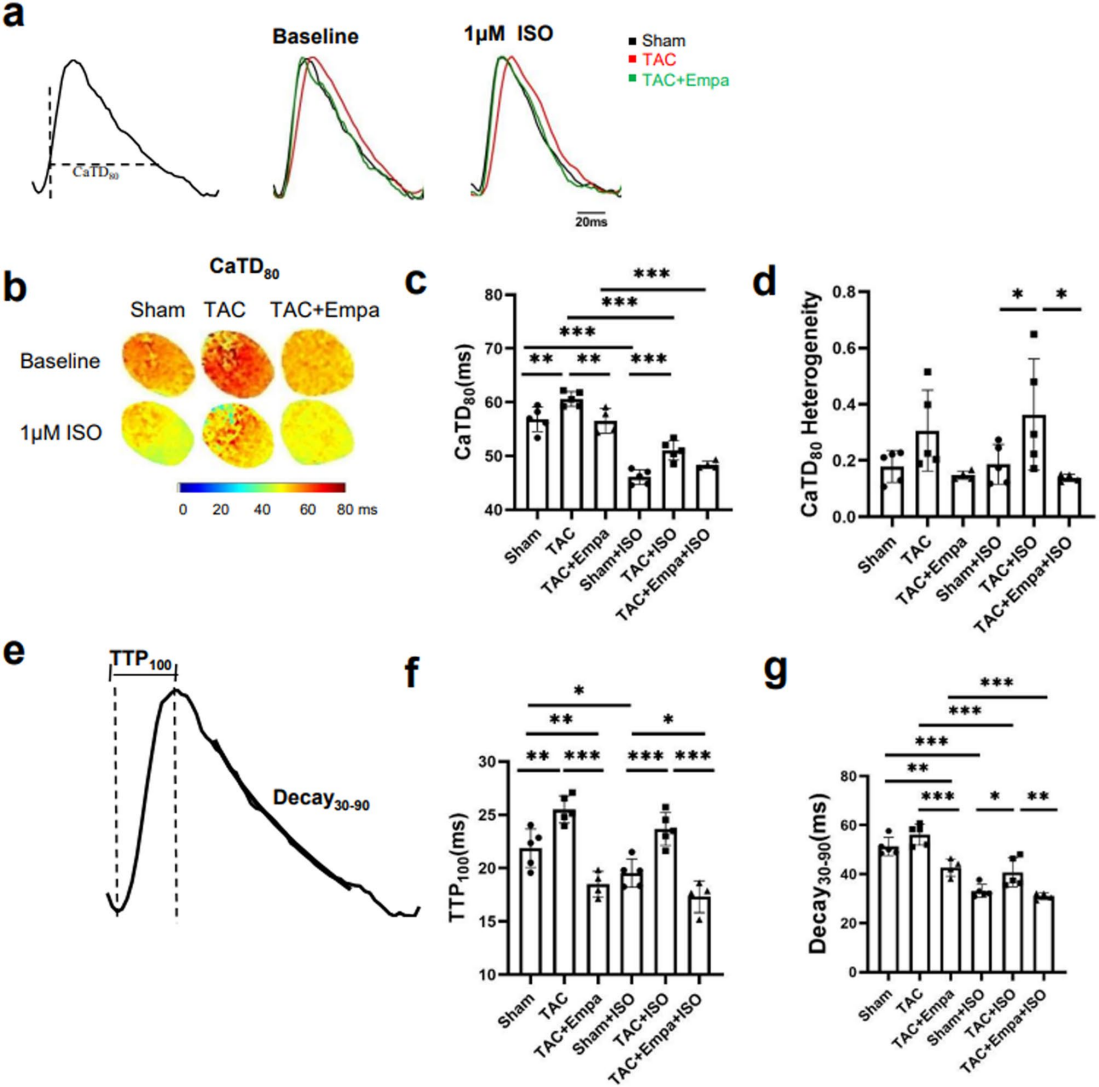
Kinetic features of Ca^2+^ transients in sham-operated, TAC-only and TAC + empagliflozin hearts. (**a**–**d**) Ca^2+^ signal (CaT) recovery properties: (**a**) CaT traces illustrating determination of CaT duration at 80% recovery CaTD_80,_ and typical baseline CaT records (middle panel) and CaT records obtained following 1 μM isoproterenol (ISO) challenge (right) from sham-operated, TAC-only and TAC + empagliflozin groups. (**b**) typical CaTD_80_ maps from sham-operated, TAC-only and TAC + empagliflozin groups. (**c**,**d**) Summarized CaTD_80_ (**c**) and heterogeneity results (**d**). N = 5 for sham-operated and TAC-only groups and N = 4 for TAC + Empagliflozin group. Scale bar = 20 ms. (e–g) Further analysis of Ca^2+^ signals into early release and late recovery phases quantified by TTP_100_ and Decay_30–90_, respectively. (**e**) illustrates determination of TTP_100_ and Decay_30–90_ from a typical CaT waveform. (**f**,**g**) TTP_100_ (**f**) and Decay_30–90_ results (**g**) for sham-operated, TAC-only and TAC + empagliflozin groups under regular 10 Hz pacing before and following 1 μM isoproterenol (ISO) challenge. N = 5 for sham-operated and TAC-only groups and N = 4 for TAC + empagliflozin group. *P < 0.05, **P < 0.01, ***P < 0.001, two way ANOVA with Holm-Sidak’s tests for post hoc multiple comparisons. TAC, transverse aortic constriction; APD, action potential duration; CaTD, calcium transient duration; Empa, empagliflozin; ISO, isoproterenol; TTP, time to peak.

Figure 6 displays kinetic measurements from the Ca^2+^ transients. Figure 6a,b displays representative CaT traces and corresponding maps of CaT duration at 80% recovery (CaTD_80_). Two way ANOVA demonstrated independent (*P* < 0.001 and *P* < 0.0001) non-interacting effects of the different interventions in the presence or absence of isoproterenol on CaTD_80_. Holm-Sidak tests revealed: (a) greater CaTD_80_ in TAC-only than sham-operated groups in both the absence or presence of isoproterenol (*P* < 0.01 and < 0.001 respectively). (b) Empagliflozin reduced this effect of TAC on CaTD_80_ before (*P* < 0.01) but not following (*P* > 0.05) isoproterenol treatment. (c) Isoproterenol reduced CaTD_80_ in all, sham-operated, TAC-only and TAC + empagliflozin groups (All *P* < 0.0001) (Fig. 6c). In contrast, two way ANOVA demonstrated independent effects (*P* < 0.05) of the different interventions but neither independent or interacting effects of isoproterenol challenge on CaTD_80_ heterogeneity (Fig. 6d). Holm-Sidak tests revealed increased heterogeneity following TAC (*P* < 0.05), restored by empagliflozin (*P* < 0.05) only in the presence of isoproterenol. Isoproterenol did not affect CaTD_80_ heterogeneity within individual treatment groups.

Further analysis of the CaT signals demonstrated that times to peak (TTP_100_) were greater in TAC-only than sham-operated hearts (Fig. 6e). They were then significantly shortened in TAC + empagliflozin hearts whether before or following isoproterenol challenge. Thus, two-way ANOVA demonstrated independent non-interacting effects of both the different interventions (*P* < 0.0001) and the presence or otherwise of isoproterenol challenge (*P* < 0.01) on TTP_100_. Holm-Sidak tests revealed: (a) greater TTP_100_ in TAC-only compared to sham-operated whether in the absence (*P* < 0.01) or presence of empagliflozin (*P* < 0.01), with empagliflozin significantly affecting results following TAC (*P* < 0.0001); (b) There were concordant significances following isoproterenol challenge (*P* < 0.001, 0.05 and 0.0001 respectively). (c) Isoproterenol itself produced significant differences only in the sham-operated group (*P* < 0.05) (Fig. 6f).

Finally, empagliflozin decreased Decay_30–90_ values quantifying the late recovery phases of the CaT transients, following TAC in both the absence and presence of isoproterenol (Fig. 6e). Thus, two-way ANOVA demonstrated independent non-interacting effects of both the different treatments (*P* < 0.01) and isoproterenol challenge (*P* < 0.0001) on Decay_30–90_ values. Holm-Sidak tests revealed: (a) reduced Decay_30–90_ in empagliflozin treated TAC compared to either sham-operated (*P* < 0.01) or TAC-only groups (*P* < 0.0001) before isoproterenol challenge. (b) There was an increased Decay_30–90_ in TAC-only compared to sham-operated groups before (*P* < 0.05) then reduced by empagliflozin treatment (*P* < 0.01). (c) Isoproterenol treatment reduced Decay_30–90_ in all, sham-operated, TAC-only or TAC + empagliflozin, groups (*P* < 0.0001, 0.0001 and 0.001) (Fig. 6g). This fulfilled established expectations from β-adrenergic receptor agonist actions of isoproterenol enhancing Ca^2+^-transporting Ca^2+^-ATPase (SERCA) function.

### Empagliflozin rescues CaT alternans associated with TAC-induced heart failure

**Figure 7:**
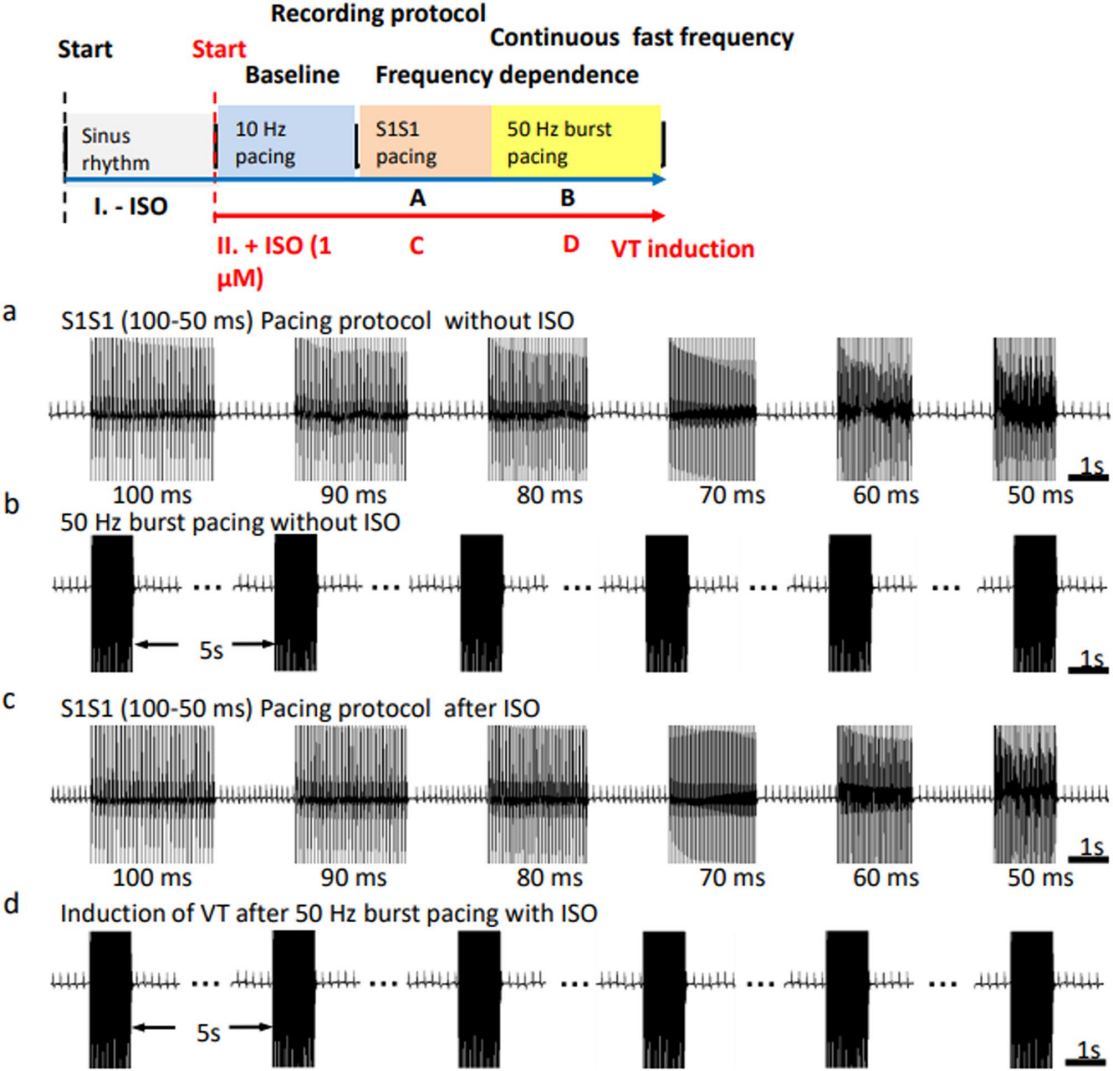
Stimulus protocols to assess ventricular arrhythmogenicity. Inset: overall pulsing scheme. (**a**) Successive episodes of 30 identical and consecutive S1 stimuli were applied with PCL decreasing from 100 to 50 ms with a 10 ms decrement. If no VT occurred during this procedure then (**b**) a burst pacing protocol consisting of 50 identical and consecutive stimuli separated by a 20 ms interval was applied. Otherwise, isoproterenol (ISO) was applied followed by similar stimulus protocols (**c**,**d**). ISO, isoproterenol; VT, ventricular tachycardia; PCL, pacing cycle length. Scale bar = 0.1 s.

**Figure 8:**
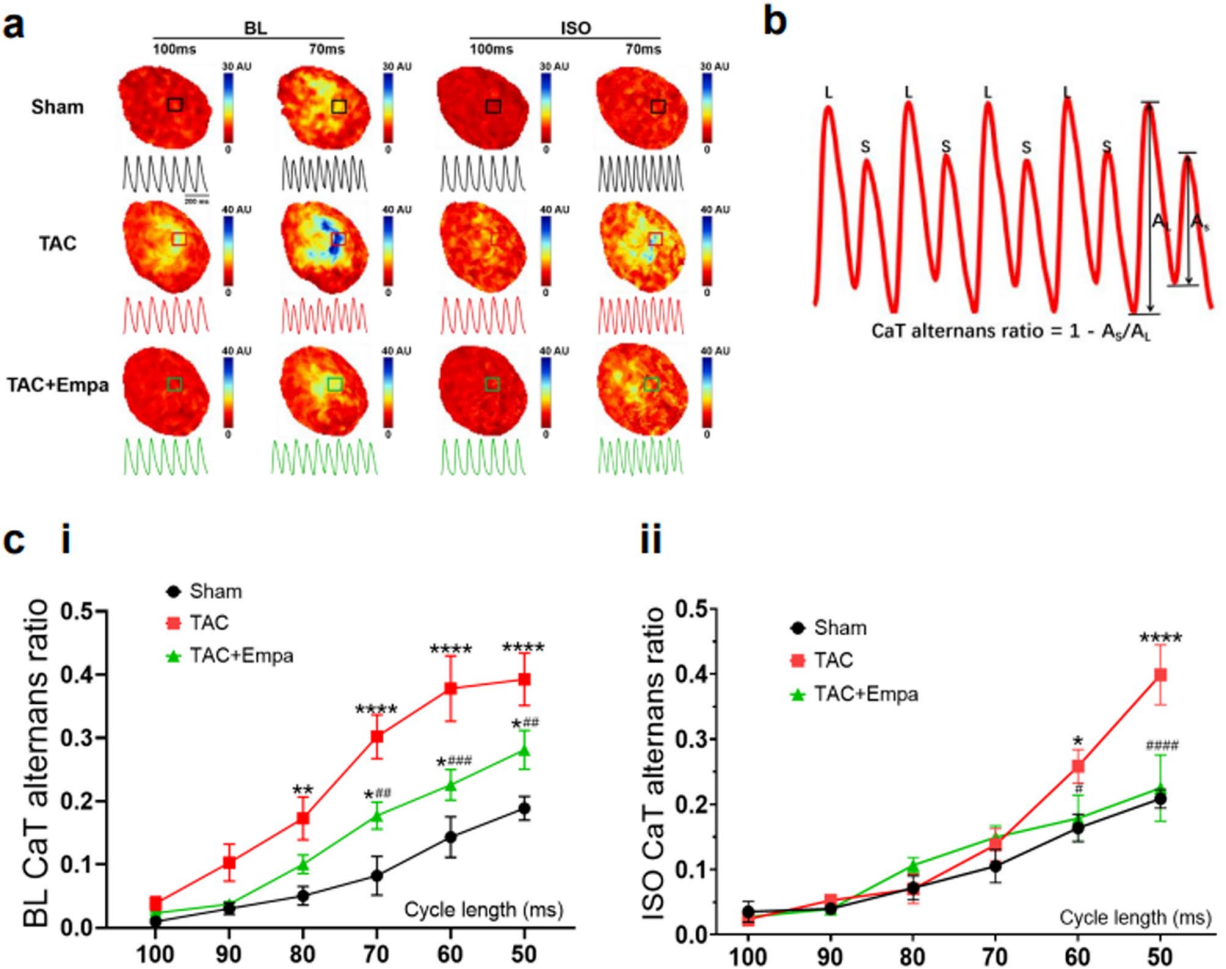
Empagliflozin blunts Ca^2+^ signal alternans induced by TAC. Analysis of CaT alternans studied in Langendorff perfused hearts analysis subject to the S1S1 protocol examining sequences containing more than 10 consecutive alternans. (**a**) Representative CaT alternans maps and traces obtained at PCLs of 70 ms and 100 ms from the sham-operated, TAC-only and TAC + empagliflozin groups before and following isoproterenol (ISO) challenge. (**b**) Plots of large and small CaT alternans and definition of CaT alternans ratio. (**c**) Quantitative plots of CaT alternans ratio at different PCLs for the sham-operated, TAC-only and TAC + empagliflozin groups before (i) and following (ii) isoproterenol challenge. For each group, N = 5 and *P < 0.05, **P < 0.01, ***P < 0.001, ****P < 0.0001 v.s sham-operated group, ^#^*P* < 0.05, ^##^*P* < 0.01, ^###^*P* < 0.001, ^####^*P* < 0.0001 v.s TAC-only group, repeated measure ANOVA with Holm-Sidak’s tests for CaT alternans ratio amongst groups at different PCLs. TAC, transverse aortic constriction; Empa, empagliflozin; ISO, isoproterenol; CaT, calcium transient; PCL, pacing length; BL, baseline.

The existence and extent of beat-to-beat CaT alternans was assessed in the Langendorff perfused hearts before and following isoproterenol challenge. A S1S1 stimulation protocol applied successive episodes of 30 identical and consecutive S1 stimuli with pacing cycle lengths (PCLs) progressively decreasing by 10 ms from 100 to 50 ms (Fig. 7a,c)**.** Figure 8a shows representative mappings of CaT alternans for all three experimental groups. Findings from anatomically equivalently and consistently positioned regions of interest selection provided corresponding alternans traces at PCLs of 100 ms and 70 ms before and following isoproterenol challenge. Such findings were quantified by a CaT alternans ratio between the CaT deflections from successive large and small CaT transients (Fig. 8b). Their pooled data (Fig. 8c) showed that CaT alternans ratio increased with PCL shortening in all groups. The TAC-only hearts showed the largest increases in CaT alternans ratio followed respectively by TAC + empagliflozin and sham-operated hearts (*P* < 0.05). Before isoproterenol challenge, CaT alternans ratio in the TAC-only and TAC + empagliflozin groups were larger than the sham-operated group for PCL < 80 ms (*P* < 0.01) and < 70 ms (*P* < 0.05) respectively. TAC-only hearts showed significantly larger CaT alternans ratios than TAC + empagliflozin hearts for PCL < 70 ms (*P* < 0.01, Fig. 8c(i)). After isoproterenol challenge, CaT alternans ratios were indistinguishable between sham-operated and TAC + empagliflozin groups at all PCLs. Ratios in TAC-only hearts differed from those of sham-operated and TAC + empagliflozin hearts when PCL < 60 ms (Fig. 8c(ii)). There were no such differences observed among groups for APD alternans with/without isoproterenol challenge.

### Empagliflozin rescues ventricular arrhythmogenicity and VT conduction patterns associated with TAC-induced HF

**Figure 9:**
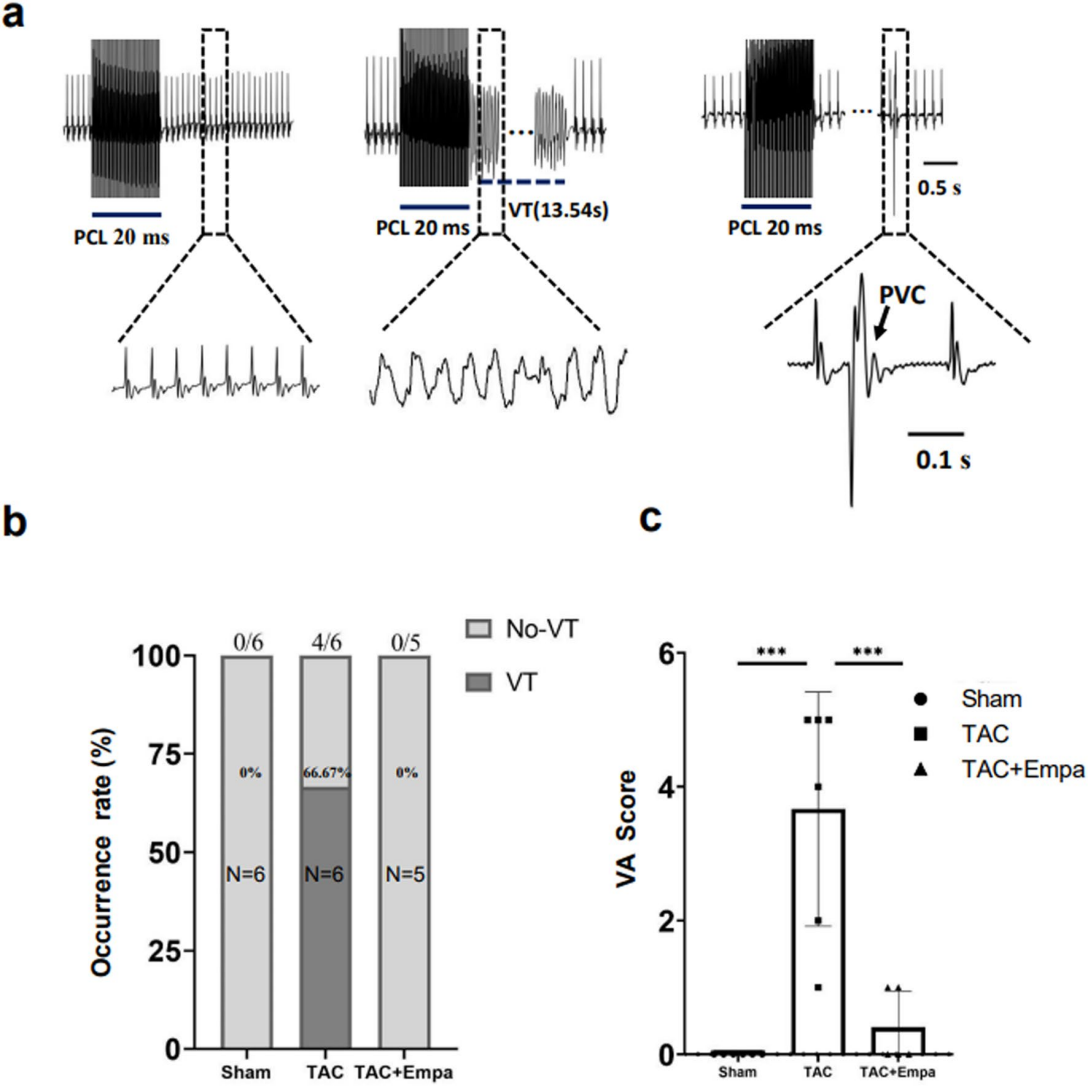
Empagliflozin rescues ventricular arrhythmogenicity following TAC. When intact Langendorff-perfused hearts were subject to S1S1 stimuli delivered at 50 Hz, no VT episodes were induced (data not shown). (**a**) After recovery, hearts were next challenged with 1 μM isoproterenol (ISO) and again subject to 50 Hz stimulation. Typical ECGs from sham-operated, TAC-only and TAC + empagliflozin (Empa) groups. Scale bar = 0.1s. PVC, premature ventricular contraction. (**b**) Incidences of VT in the three experimental groups giving total numbers of hearts studied, and the numbers of hearts showing VT in each group. (**c**) Scoring of occurrences of ventricular arrhythmogenicity in sham-operated, TAC-only and TAC + empagliflozin groups.

Arrhythmic tendency was assessed before and following isoproterenol challenge. Under each condition, the initial pulse protocol comprised successive episodes of 30 identical and consecutive S1 stimuli with PCL decreasing from 100 to 50 ms in 10 ms decrements (Fig. 7a,c). If no VT occurred during this procedure, a burst pacing protocol comprising 50 identical and consecutive stimuli separated by 20 ms intervals was applied (Fig. 7b,d). Before isoproterenol challenge all three experimental groups showed no arrhythmic effects with either S1S1 or burst pacing. Following 1 μM isoproterenol challenge, burst but not S1S1 pacing induced VT episodes in only the TAC-only hearts. Figure 9a illustrates ECG recordings of normal activity, VT and premature ventricular contractions. Figure 9b summarizes incidences of VT in the three experimental groups giving total numbers of hearts studied, and the numbers of hearts showing VT in each group. Hearts in the TAC only, but not the sham-operated or TAC + empagliflozin groups showed occurrences of VT. Finally, a simple scheme scored occurrences of ventricular arrhythmogenicity, as an absence of arrhythmias (score = 0), occurrence of isolated (score = 1), bigeminy or frequent (> 10/min) (score = 2), paired premature ventricular contractions (PVCs) (score = 3), and nonsustained (score = 4) and sustained VT lasting > 2 s (score = 5). This gave significantly higher scores in the TAC-only than the other two groups (22 vs. 0 vs. 2 for the TAC-only, sham-operated and TAC + empagliflozin group, *P* < 0.001, Fig. 9c).

**Figure 10:**
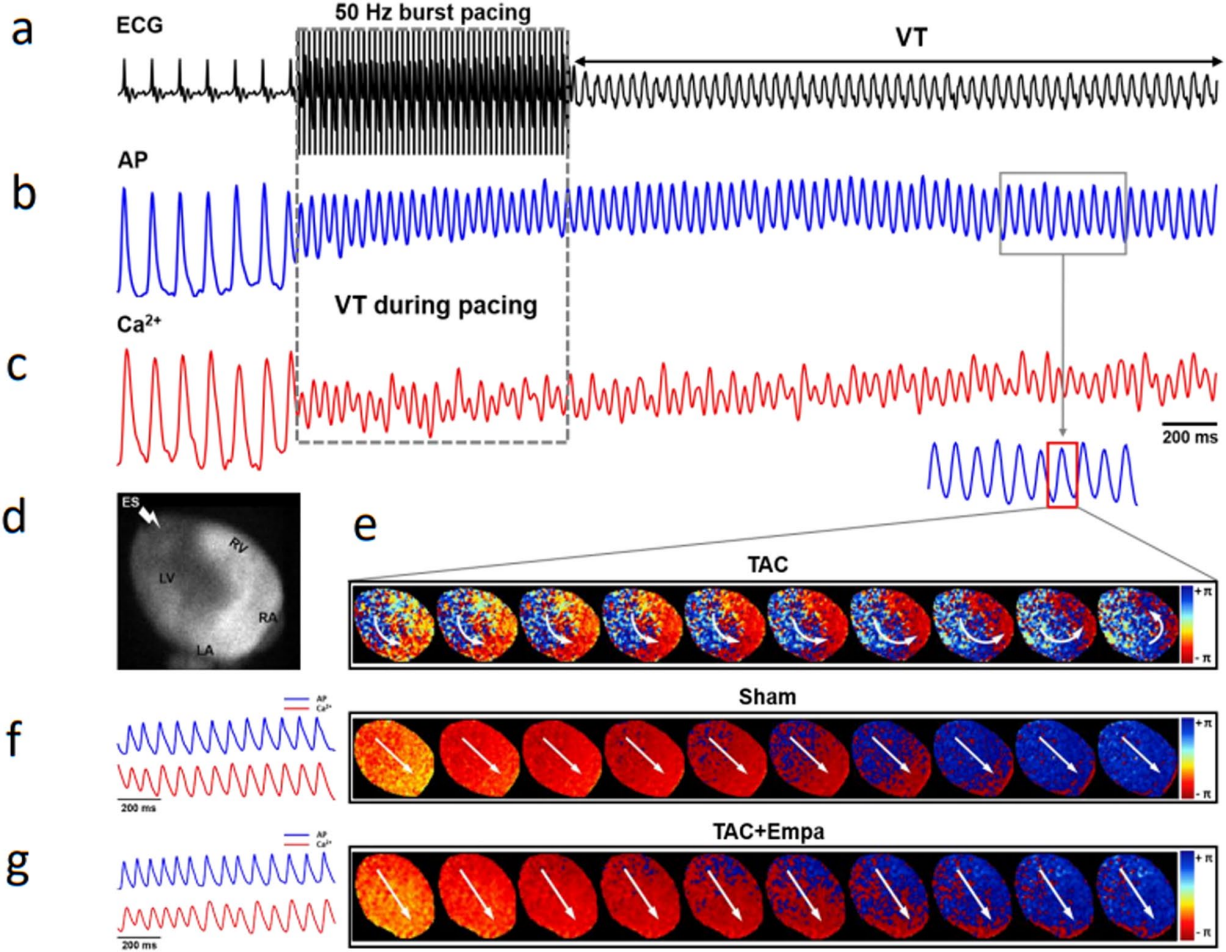
Conduction patterns during a ventricular tachycardic (VT) episode in a heart following TAC induced HF. (**a**–**c**) Simultaneous ECG (**a**), membrane potential, *V*_m_ (**b**) and Ca^2+^ recordings (**c**) close to the onset of VT obtained for phase mapping analysis for conduction made over the region of interest shown in the inset (**d**) giving representative maps of macro-reentry at different time points in the TAC-only group (**e**). No VT episodes were observed in sham-operated (**f**) and TAC + empagliflozin (**g**) groups which gave rectilinear conduction patterns running from the stimulus point at the apex to the base. TAC, transverse aortic constriction; Empa, empagliflozin; *V*_m_, membrane potential.

VT episodes were studied using simultaneous ECG, AP and CaT recordings (Fig. 10a–c) then examined by phase analysis over a selected region of interest (Fig. 10d) and real-time videos of activation (Supplementary File S3). They only occurred in the TAC-only group following 1 μM isoproterenol with burst pacing at 50 Hz. They resulted in re-entrant conduction patterns (Fig. 10e) through successive time points. Supplementary File S3 illustrates the results of an OMapScope 5 v5.7.8 phase mapping analysis to detect and characterize re-entrant activity during sustained arrhythmia. This yielded a centered macro re-entry conduction pattern demonstrated close to the onset of ventricular tachycardia. The remaining sham-operated and TAC + empagliflozin hearts did not show VT, consistently displaying only regular action potential and CaT traces and linear wavefront conduction patterns (Fig. 10f,g).

## Discussion

SGLT2i agents hold future promise for the clinical management of heart failure (HF). We here complement previous *cellular-level* studies reporting effects of the selective SGLT2i empagliflozin following TAC-induced HF and hypoxic stress on isolated cardiomyocytes, proceeding to study its effects on *intact hearts*. The previous reports had demonstrated SGLT2i actions on an altered pro-arrhythmic *I*_NaL_^20,22^, Ca^2+^ homeostasis and CaMKII activity^13,22^ in isolated and/or iPSC-derived ventricular cardiomyocytes. We now explore corresponding anatomical and systolic changes, related alterations in electrophysiological action potential properties, Ca^2+^ homeostasis and CaMKII phosphorylation, and the consequent arrhythmic tendency at the level of intact TAC-HF hearts for the first time. The use of intact TAC-HF hearts here also complements previous experimental cellular and whole heart studies of empagliflozin effects on these parameters under alternative conditions of diabetes, metabolic deficiency and ischemia. These had similarly examined electrophysiological properties including *I*_NaL_^8^, diastolic function^11,12^, and CaMKII phosphorylation^11^.

Our initial experiments compared some specific actions of empagliflozin obtained under our present experimental conditions to reports from previous cellular level studies. First, earlier studies had reported that empagliflozin decreased *I*_NaL_ in patch-clamped ventricular cardiomyocytes from diabetic rats^8^ and TAC mice^20^. Docking modelling studies had attributed this action to its binding to Nav1.5 domains III and IV^20^, in common with binding by the specific *I*_NaL_ inhibitor ranolazine. We confirmed such effects specifically for expressed cardiac Nav1.5: empagliflozin reduced *I*_NaL_ whilst sparing Na^+^ peak current activation and inactivation gating properties. Second, in previous studies, empagliflozin reversed the up-regulated RyR2 phosphorylation attributable to the increased CaMK-II activity in ventricular cells from db/db and TAC-HF mice and human failing hearts that could perturb Ca^2+^ homeostasis^7,11,13^. A similar finding was confirmed in our western blot results examining p- and t-CaMK-II in our sham-operated, TAC-only and TAC + empagliflozin hearts. Third, the related SGLT2i dapagliflozin ameliorated elevations of Nav1.5 protein expression in a high salt induced hypertension rat model^19^. We here confirmed an elevated Nav1.5 protein expression downregulated by empagliflozin treatment in the present murine TAC-induced HF model for the first time. Finally, whereas empagliflozin increased reverse NCX potentially increasing intracellular Ca^2+^ overload, in ventricular myocytes from diabetic rats^8^, it did not do so in cardiomyocytes from mice with heart failure^20^. Our present findings do not report empagliflozin actions on NCX expression that could otherwise influence the occurrence or otherwise of Ca^2+^ overload^23^.

The present studies on whole Langendorff-perfused hearts then examined effects of chronic empagliflozin challenge. Comparing the TAC-only and sham-operated hearts confirmed physiological and anatomical features associated with heart failure. This took the form of reduced, echocardiographically measured, ejection fractions and fractional shortenings, and increases in diastolic anterior and posterior wall thicknesses. The TAC + empagliflozin experimental groups then showed an amelioration of these effects.

Electrophysiological, Ca^2+^ homeostatic changes and pro-arrhythmic changes in the intact hearts could then be matched to these echocardiographic and histological findings. Firstly, dual optical voltage recordings demonstrated TAC-induced increases in action potential durations at 80% recovery (APD_80_) measured in regularly (10 Hz) paced hearts whether in the presence or absence of isoproterenol. These were ameliorated by empagliflozin before though not following isoproterenol challenge. These findings parallel the Nav1.5 effects upon *I*_NaL_ and upon Nav1.5 protein expression. APD_80_ heterogeneity was not altered through manoeuvres involving TAC with or without empagliflozin challenge, and whether isoproterenol was present or absent.

Secondly, dual optical Ca^2+^ transient mapping demonstrated increased Ca^2+^ transient (*F*/*F*_0_) peak magnitudes and times to peak Ca^2+^ (TTP_100_), durations at 80% recovery (CaTD_80_) and Ca^2+^ decay constants (Decay_30–90_) during regular, 10 Hz, stimulation in the TAC-only compared with sham-operated hearts, whether isoproterenol was present or absent. It also demonstrated Ca^2+^ transient alternans with shortening stimulus cycle length. The actions on *F*/*F*_0_, TTP_100_ and Decay_30–90_ were again relieved in the TAC + empagliflozin hearts both in the absence and presence of isoproterenol; those on CaTD_80_ were relieved before but not following isoproterenol challenge.

Comparing the findings bearing on Ca^2+^ homeostasis with the echocardiographic changes also reported here suggests partial compensation of the heart to the reduced ejection fractions and fractional shortening indicated above. However, TAC also produced morphological changes, reflected in the observed cardiac hypertrophy with increased diastolic anterior and posterior wall thicknesses and heart weight/tibial length ratios. These could potentially produce the compromised overall cardiac contractility at the whole heart level previously associated with hypertrophic change. The dual mapping also demonstrated Ca^2+^ transient alternans with shortening stimulus cycle length. These actions were again relieved in the TAC + empagliflozin hearts*.* The findings thus add to single cardiomyocyte results in empirically clarifying whole heart actions of empagliflozin on Ca^2+^ homeostasis.

Thirdly, isoproterenol challenge then shortened APD_80_ in the sham-operated and TAC-only hearts. It increased *F*/*F*_0_, shortened both CaTD_80_ and Decay_30–90_ in all sham-operated, TAC-only and TAC + empagliflozin groups. In contrast, it shortened TTP_100_ in sham-operated but spared TTP_100_ in TAC-only and TAC + empagliflozin groups. It spared Ca^2+^ transient alternans in all groups. These findings are consistent with its established actions on Ca^2+^-ATPase through phospholamban phosphorylation. APD_80_ heterogeneities remained similar in all groups but CaTD_80_ heterogeneities were greater in the TAC-only hearts, whether before or following isoproterenol challenge.

Previous *cellular level* findings in ventricular cells from db/db and TAC-HF mice and failing human hearts confirm parallel alterations in CaMKII expression potentially increasing RyR2 phosphorylation and activity^8,11,13^. The present studies reported an empagliflozin rescue of a prolonged Decay_30–90_ that might reflect SR Ca^2+^ re-uptake. However, reports of its potential actions on SERCA processes are more varied. It increased PLN serine and threonine site phosphorylation whilst decreasing t-PLN in ob/ob mice^16^, but decreased p-PLN (at threonine 17) sparing t-PLN expression in failing hearts from TAC mice^13^. It rescued a compromised SERCA protein expression in streptozotocin-induced diabetic rat ventricular cells^8^ but did not affect such expression in ventricular cells of TAC-HF^13^, ob/ob^16^ or db/db-induced diabetic mice, and deoxycorticosterone acetate salt-induced rats with heart failure with preserved ejection fraction (HFpEF)^11,21^.

Fourthly, the experiments, in any event, also demonstrated that empagliflozin rescued instabilities in Ca^2+^ homeostasis following TAC in intact hearts paced at progressively decreasing PCL. These instabilities were quantified using CaT alternans ratios between maximum CaT deflections of successive, alternating, large and small CaT transients. Although such ratios increased with PCL shortening in all groups, this was most marked in TAC-only, followed by TAC + empagliflozin and sham-operated hearts (*P* < 0.05). With isoproterenol challenge, the greater ratios persisted in the TAC-only hearts and were similar between sham-operated and TAC + empagliflozin groups.

Finally, burst, but not regular S1S1 pacing at progressively increased frequencies, induced VT only in TAC-only but not the remaining sham-operated or TAC + empagliflozin hearts doing so only following pro-arrhythmic 1 μM isoproterenol challenge. With the VT episodes, re-entrant mapping patterns on re-entrant analysis replaced the normal regular wavefront propagation of excitation.

Together these findings in the murine TAC experimental platform thus complement experimental studies in diabetic rats, high-fat-diet db/db mice and myocardial ischemic models. They further extend those earlier cellular level studies using the TAC experimental platform in isolated and or iPSC-derived ventricular cardiomyocytes bearing on APD^7,8^ and *I*_NaL_^8,20,22^, and alterations in Ca^2+^ homeostasis^8,11,12^ reflecting CaMK-II mediated^11,13^ actions on Ca^2+^ signaling proteins including ryanodine receptor type II (RyR2) and phospholamban (PLN)^8,11,12,14,15^. Such cellular parameters translate to the level of intact hearts, in the form of the empagliflozin-induced rescues of compromised systolic function, ventricular hypertrophy, electrophysiological and intracellular Ca^2+^ homeostatic changes, and arrhythmogenicity.

## Materials and methods

### TAC-induced heart failure model

All research protocols were approved by the Institutional Animal Care and Use Committee of Southwest Medical University, and all experiments were performed in accordance with its relevant guidelines and regulations and conformed to ARRIVE guidelines. In common with a substantial proportion of previous reports^24^, 8 week old male C57BL6/J mice (Chongqing Tengxin Biotechnology Co., Ltd, China), weight ~ 25 g were housed in a temperature- and humidity-controlled animal facility with 12:12 h light/dark cycles with water ad libitum. TAC was performed to create a pressure overload-induced heart failure as described previously^25^. Briefly, mice were anesthetized with 5% isoflurane. On reaching a surgical plane, mice were fixed in a supine position with medical tape, and anesthesia was maintained with 1.5–2% isoflurane. The neck incision under the thyroid gland was made extending to the sternum handle. The muscles were separated to expose the trachea and the right common carotid artery. This was followed down to the aortic arch. The transverse arch was carefully dissected free of connective tissue and a 6/0 silk tie was passed around the arch between the innominate and left carotid arteries. A double blunted 27G (0.4 mm) needle was then placed alongside the arch and the tie ligated. The blunted needle was then removed to standardize the stenosis. The thymus was gently replaced, and retractors removed, the sternum and skin incision were closed successively using 6/0 vicryl suture. Sham operated animals underwent the same procedure without banding. When the animals had regained consciousness and were moving around, bedding, food and water were then provided ad libitum. There were three experimental groups. Of these, a sham-operated group did not undergo any TAC procedure or empagliflozin (Targetmol, Wellesley Hills, MA, USA; #T1766) challenge. This was studied 6 weeks following the start of the protocol. The TAC-only group underwent the TAC procedure and was studied 6 weeks postoperatively. The TAC + empagliflozin group underwent the TAC procedure; from 2 weeks after that, it underwent a 4 week period of empagliflozin application, for study similarly at 6 weeks postoperatively. This mimicked a clinical situation when empagliflozin treatment might commence or otherwise a period following onset of the underlying pathology.

### Single-cell electrophysiological recordings

Whole-cell-patch-clamp Na^+^ currents were recorded from CHO cell lines (Henan Scope Research Institute of Electrophysiology) stably expressing human Nav1.5 α-subunits. Cells were maintained in T-25 cell culture flasks containing DMEM complete medium supplemented with 10% fetal bovine serum, 1% penicillin–streptomycin and geneticin (G418, 400 μg/mL) at 37°C under 5% CO_2_. Cells were passaged using trypsin and plated onto small sterilized glass cover slips in 35-mm Petri dishes with complete DMEM. Cells were then incubated for 12–24 h prior to electrophysiological study. Currents were recorded using a MultiClamp 700A amplifier, 1550 A digitizer and pCLAMP 10.6 software (Molecular Devices, San Jose, CA, USA) at room temperature (22–24 °C), low-pass filtered at 6 kHz and digitized at a 5 kHz sampling rate (Digidata700, Axon Instruments Inc). Patch electrodes with resistances of 1–3 MΩ were pulled with a P-97 micropipette puller (Sutter Instruments, Novato, CA, USA). The external solution contained (mM): NaCl 137; MgCl_2_ 1; KCl 4; glucose 10; HEPES 10; CaCl_2_ 1.8, pH adjusted to 7.4 with NaOH. The sodium current pipette solution contained (mM): CsCl 65; CsF 75; MgCl_2_ 2.5; EGTA 5; HEPES 10, pH adjusted to 7.3 with CsOH. Na^+^ currents were elicited by 100 ms voltage steps to − 15 mV from a holding potential of − 120 mV with a frequency of 0.5 Hz. *I*_NaL_ was enhanced by sea anemone toxin II (ATX-II cat. no. ab141870, Abcam, UK).

### Western blot studies

50 μg total protein extracted from whole heart tissue for each lane was separated using 5% stacking gel and 10% separation gel and transferred to a 0.45 μm polyvinylidene fluoride membrane (Millipore, Burlington, MA, USA). The membrane was incubated in TBST containing 5% nonfat milk for 2 h at room temperature to block nonspecific binding and was incubated with the primary antibody overnight at 4 °C. The primary antibodies for phosphorylated CaMKII (p-CaMKII) (T287) (cat. no. ab182674, Abcam, Cambridge, UK, 1:1000), total CaMKII (t-CaMKII) (cat. no. ab22609, Abcam, Cambridge, UK, 1:1000), and the internal control antibody GAPDH (Santa Cruz Biotechnology, Santa Cruz, CA, USA, 1:1000) were used. The membrane was incubated with the goat anti-mouse IgG HRP (BBI Life Sciences, China) and goat anti-rabbit IgG HRP (BBI Life Sciences, China) secondary antibody (1:1000) for 1 h at room temperature. The membrane was incubated in chemiluminescent HRP substrate (Millipore, Burlington, MA, USA) at room temperature for 5 min and then imaged with the Universal Hood II System (Bio-Rad, Hercules, CA, USA).

### Echocardiographic and hemodynamic analysis in anaesthetized animals

To confirm the presence or otherwise of heart failure, at two weeks post-surgery, animals were lightly anesthetized with 1.5–2% isoflurane using a gas anesthesia machine (RWD Life Science, Shenzhen city, Guangdong province, China). The chest was shaved and a 30 MHz Doppler probe attached to the 3100 Vevo Preclinical Imaging System (FUJIFILM Visual Sonics, Toronto, Canada) was used to measure the blood flow velocity (mm/sec) across the aortic constriction. Mice in the sham-operated and TAC-only group were administrated 0.9% normal saline by daily gavage while those in the TAC + empagliflozin group received 10 μM empagliflozin over a 4 week period. All mice were then investigated by trans-thoracic M-mode echocardiography along the parasternal short axis in the supine, and the parasternal long axis in the left lateral positions respectively to derive cardiac size and aortic parameters. These yielded the cardiac systolic function parameters of ejection fraction (EF), fractional shortening (FS) and structural parameters of the left ventricular systolic and diastolic/posterior wall thicknesses and internal diameters. Mice were then sacrificed for electrophysiological and biochemical studies. Body and heart weight and tibia length were also recorded.

### Optical mapping of membrane potential and Ca^2+^ signals in Langendorff perfused hearts

Electrophysiological function and arrhythmic susceptibilities of ex vivo intact hearts were assessed using the custom-designed optical mapping system equipped with an EMCCD camera (Evolve 512 Photometrics, Tucson, AZ, USA) described previously^26,27^. Briefly, mice were anesthetized with 2–5% isoflurane. This was followed by an intraperitoneal injection of 100 units heparin. Following cervical dislocation, hearts were rapidly excised and Langendorff-perfused with oxygenated physiological salt solution (PSS) (containing in mM: NaCl 119, NaHCO_3_ 20, NaH_2_PO_4_ 1.2, KCl 4.7, MgCl_2_ 1.0, CaCl_2_ 1.3, and glucose 10; equilibrated with 95% O_2_ and 5% CO_2_, pH 7.4) at 37 °C and at a constant 2–3 ml/min flow rate for 10 min. The Ca^2+^ dye Rhod-2 AM (Thermo Fisher Scientific, UK) was loaded as a 50 μl bolus (stock solution: 1 mg/ml in DMSO) and re-circulated for 20 min in the presence of 0.5 mM Pluronic F127. The voltage-sensitive dye RH237 (Thermo Fisher Scientific, UK) was loaded as a 10 μl bolus of concentration 1 mg/ml over a 10 min period. After dye loading, hearts were perfused with PSS solution containing 10 μM blebbistatin (MedChemExpress, Monmouth, Junction, NJ, USA), a myosin II inhibitor used to inhibit contractions and prevent movement artefacts during optical mapping recordings.

Optical electrophysiological assessments of relative [Ca^2+^]_i_ and voltage changes used four 530 nm LEDs for the Rhod-2 and RH237 excitation. Ca^2+^ transient (CaT) signals were collected at a 585 ± 40 nm wavelength while membrane potential (*V*_m_) signals were collected using a 662 nm long pass filter. The maximum camera 512 × 512 pixel^2^ area was 2-binned to optimize sampling frequency, giving a 256 × 256 pixel^2^ spatial representation of which the cardiac mapping area occupied an 83 × 83 pixel^2^ acquisition region. This permitted the acquisitions of 4500 frames over 7.959 s at sampling frequency 0.565 kHz (4500/7.959 = 565 Hz) and exposure time 0.9 ms (Fig. 5a). The work distance setting between camera and entire heart provided a distance scale of 242 × 242 μm^2^/pixel. Readings were obtained over a S1S1 stimulation protocol comprising 30 consecutive electrical stimulations at a 100 ms PCL. Recorded image files were loaded into ElectroMap optical mapping analysis software^28^. All signals then underwent spatial, 3 × 3 pixel, Gaussian, σ = 1.5, and temporal, 3rd order Savitzky-Golay, filtering (Figs. 5, 6). Results for each pixel were then averaged for the 30 consecutive stimuli. Then, the averaged results for each pixel were averaged to obtain the mean result over the entire acquisition region. This provided action potential durations from peak to 80% recovery (APD_80_), CaT durations at 80% recovery (CaTD_80_), times to peak (TTP_100_) and decay constants of the late absorption phases of the CaT transients (Decay_30–90_). OMapScope 5 v5.7.8 software was used to obtain peak CaT values *F* normalized to baseline values *F*_0_, *F*/*F*_0_, conduction and Ca^2+^ alternans maps. Although baseline *F*_0_ values showed little variation through each experiment, and did not show significant differences between hearts within each experimental group, differences were apparent between mean *F*_0_ between groups on one way ANOVA; these would be corrected for in the *F*/*F*_0_ ratios. It was also used in phase mapping analysis for the detection and characterization of re-entrant activity associated with sustained arrhythmia^29^. This approach, previously demonstrated applicable in murine hearts^30^, permits further analysis^31^. This examined all the pixels in the field of view of successive maps of membrane voltage obtained through time^32^. In each pixel, the periodic variation of membrane voltage with time yielded an instantaneous phase value computable by Hilbert transformation, within the interval [− π, + π], at any given time *t*. Mapping of the phases at successive times would then depict the spatiotemporal changes in activation whether during normal or arrhythmic activity, and permitting detection or otherwise of re-entrant activity.

### Arrhythmia inducing protocols in Langendorff perfused hearts

Assessments for arrhythmogenicity and frequency-dependent Ca^2+^ alternans both first delivered a series of 30 regular (S1S1) stimuli at constant pacing cycle length (PCL) at the ventricular apex each separated by 2 s intervals successively decremented by 10 ms, between PCLs of 100 and 50 ms. If VT was then not induced the arrhythmogenicity assessments then further applied burst pacing protocols of 50 consecutive pulses at a 20 ms PCL assessed for VT inducibility. VT was defined as an occurrence of 3 consecutive regular abnormal waveforms, and results were scored for pro-arrhythmic tendency. Experiments were performed both before and following 1 μM isoproterenol (cat: HY-B0448, MedChemExpress, Monmouth Junction, NJ, USA) challenge to increase arrhythmic susceptibility.

### Statistical analysis

Patch-clamp recorded data were analyzed using Clampfit 10.6 (Molecular Devices, USA), OriginPro 8.0 (Origin Lab, Northampton, USA) and Adobe Illustrator 10 (Adobe, USA). All data are expressed as mean ± SEM or medians with interquartile range based on data distribution. Normality and variance equality were tested by the Shapiro–Wilk and Brown–Forsythe tests, respectively. One- or two-way ANOVA followed by post hoc Holm-Sidak’s multiple-comparison or Kruskal–Wallis followed by post hoc Dunn’s multiple-comparison test were used as appropriate for continuous and discontinuous variable analysis among groups, respectively. Detailed statistical methods are described in the Figure legends. All parametric and non-parametric tests were two-sided, with a significance level set at 5%.

### Supplementary Information

Supplementary Figure 1.

Supplementary Figure 2.

Supplementary Video 1.

Supplementary Information 1.

## Data Availability

The datasets used and/or analysed during the current study available from the corresponding author on reasonable request. Supplementary Files S2 and S3 are respectively available on Figshare, under the following citations: Ou, Xianhong (2023): Original WB. figshare. Figure. 10.6084/m9.figshare.23254775.v1. Ou, Xianhong (2023): Reentry video of heart of TAC mouse. figshare. Media. 10.6084/m9.figshare.23254790.

## Abbreviations

APD: Action potential duration
APD_80_: Action potential duration at 80% recovery
ATX-II: *Anemonia sulcata* Toxin II
BL: Baseline.
CaT: Ca^2+^ transient
CaTD: Calcium transient duration
CaTD_80_: Ca^2+^ transient duration at 80% recovery
CV: Conduction velocity
Decay_30–90_: Ca^2+^ transient decay time
ECG: Electrocardiography
EF: Ejection fraction
Empa: Empagliflozin
*F*: Fluorescence signal
*F*/*F*_0_: *F* Normalized to baseline signal *F*_0_
*F*_0_: Baseline fluorescence signal
FS: Fractional shortening
GAPDH: Glyceraldehyde-3-phosphate dehydrogenase
HF: Heart failure
HW/TL: Heart weight/tibial length ratio
*I*_NaL_: Late Nav1.5 current
ISO: Isoproterenol
LVAW: Left ventricular anterior wall
LVID: Left ventricular internal diameter
LVPW: Left ventricular posterior wall
Nav1.5: Sodium channel 1.5
NCX: Na^+^/Ca^2+^ exchanger
p-CaMK-II: Phosphorylated calmodulin-dependent protein kinase II
PCL: Pacing cycle length
PLN: Phospholamban
PVC: Premature ventricular contraction
RyR2: Ryanodine receptor type 2
SGLT2i: Sodium-glucose co-transporter 2 inhibitor
t-CaMK-II: Total calmodulin-dependent protein kinase II
t/p-CaMK-II: Total/phosphorylated calmodulin-dependent protein kinase II
TAC: Transverse aortic constriction
TTP: Time to peak.
VA: Ventricular arrhythmia.
*V*_m_: Membrane potential
VT: Ventricular tachycardia
