# Supplementary material for: Empagliflozin rescues pro-arrhythmic and Ca2+ homeostatic effects of transverse aortic constriction in intact murine hearts
Source: Sci Rep. 2024 Jul 8;14:15683. doi: 10.1038/s41598-024-66098-7 (PMC11231339; doi:10.1038/s41598-024-66098-7)
Supplement: Supplementary file 1 — Supplementary Figure 1. [file 41598_2024_66098_MOESM1_ESM.pdf]

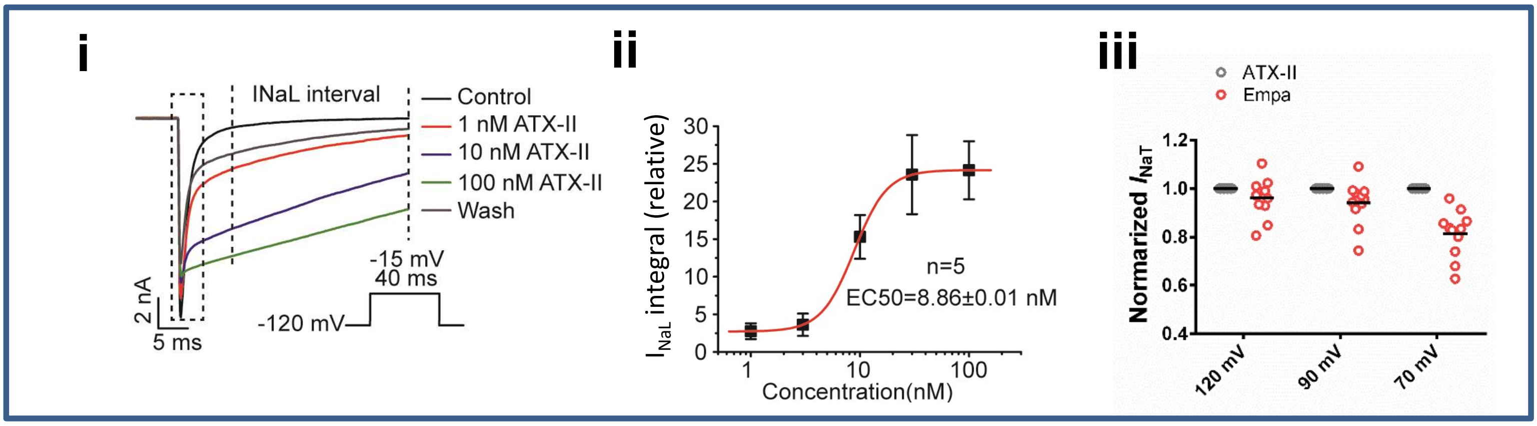

Supplementary Figure S1.

Whole cell patch clamp results in CHO cell lines stably expressing human Nav1.5 channel α subunit. Results of increasing ATX-II concentrations on Na^+ currents (i) yielding effects on late Na^+ current (I_{NaL}), measured as the area between the baseline and current trace in the 10-40 ms interval following the onset of voltage steps from the -120 mV resting to a -15 mV membrane potential. Effects of ATX-II concentrations on I_{NaL} measured relative to values obtained in the absence of ATX-II giving an EC_{50} of 8.86±0.01 nM (n = 5) (ii). Note absence of changes in initial, peak Na^+ current I_{NaT} whether in the presence or absence of ATX II or empagliflozin (Empa) (iii)
