# Supplementary material for: Empagliflozin rescues pro-arrhythmic and Ca2+ homeostatic effects of transverse aortic constriction in intact murine hearts
Source: Sci Rep. 2024 Jul 8;14:15683. doi: 10.1038/s41598-024-66098-7 (PMC11231339; doi:10.1038/s41598-024-66098-7)

Supplementary file 2.

data related to figure 2.

Original Western blot results: expression of Nav1.5, NCX, p-CaMK-II and t-CaMK-II (left) and their GAPDH references (right).

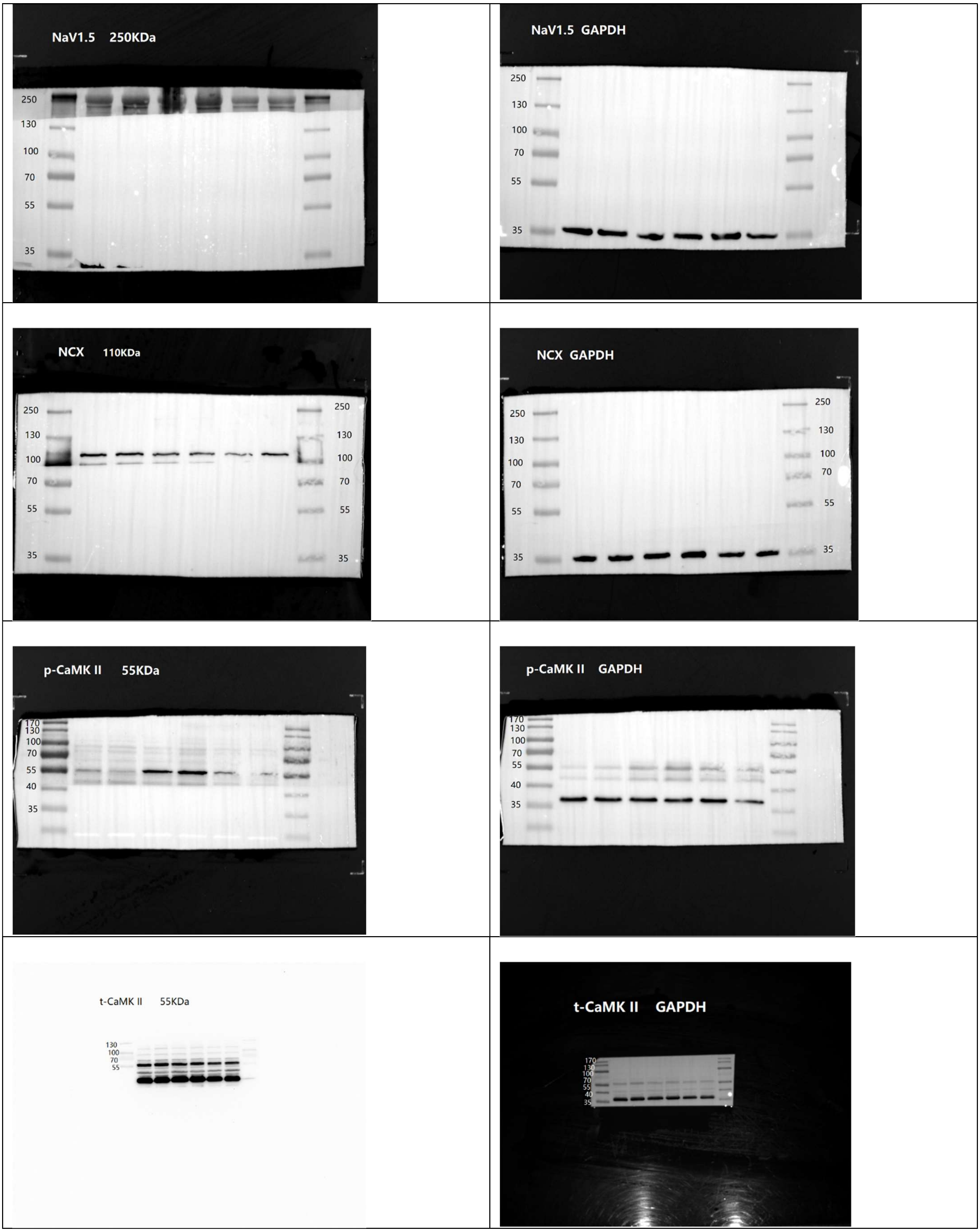

Supplement: Supplementary file 2 — Supplementary Figure 2. [file 41598_2024_66098_MOESM2_ESM.pdf]
