# Supplementary material for: Empagliflozin rescues pro-arrhythmic and Ca2+ homeostatic effects of transverse aortic constriction in intact murine hearts
Source: Sci Rep. 2024 Jul 8;14:15683. doi: 10.1038/s41598-024-66098-7 (PMC11231339; doi:10.1038/s41598-024-66098-7)
Supplement: Supplementary file 4 — Supplementary Information 1. [file 41598_2024_66098_MOESM4_ESM.docx]

**Supplementary files.**

**Supplementary file 1.**

**Supplementary file 2.**

Also available at: Ou, Xianhong (2023): Original WB. figshare. Figure. [https://doi.org/10.6084/m9.figshare.23254775.v1](https://eur03.safelinks.protection.outlook.com/?url=https%3A%2F%2Fdoi.org%2F10.6084%2Fm9.figshare.23254775.v1&data=05%7C01%7Cclh11%40universityofcambridgecloud.onmicrosoft.com%7C49e87098f165459291fd08dbd62a90d0%7C49a50445bdfa4b79ade3547b4f3986e9%7C1%7C0%7C638339250544116501%7CUnknown%7CTWFpbGZsb3d8eyJWIjoiMC4wLjAwMDAiLCJQIjoiV2luMzIiLCJBTiI6Ik1haWwiLCJXVCI6Mn0%3D%7C3000%7C%7C%7C&sdata=el7zavGJ3dGPhbiehH2ZMJusMZViePMOPbH%2FPd4Zrr0%3D&reserved=0)

**Supplementary file 3: mp4 file:** *TAC.mp4*

Also available at: Ou, Xianhong (2023): Reentry video of heart of TAC mouse. figshare. Media. [https://doi.org/10.6084/m9.figshare.23254790](https://eur03.safelinks.protection.outlook.com/?url=https%3A%2F%2Fdoi.org%2F10.6084%2Fm9.figshare.23254790&data=05%7C01%7Cclh11%40universityofcambridgecloud.onmicrosoft.com%7C49e87098f165459291fd08dbd62a90d0%7C49a50445bdfa4b79ade3547b4f3986e9%7C1%7C0%7C638339250544116501%7CUnknown%7CTWFpbGZsb3d8eyJWIjoiMC4wLjAwMDAiLCJQIjoiV2luMzIiLCJBTiI6Ik1haWwiLCJXVCI6Mn0%3D%7C3000%7C%7C%7C&sdata=XNMhyNpX7YJb7%2BhylTWSrpbYCaOOw57AhesP3lPRPa0%3D&reserved=0)
